# PRDE-1 is a nuclear factor essential for the biogenesis of Ruby motif-dependent piRNAs in *C. elegans*

**DOI:** 10.1101/gad.238105.114

**Published:** 2014-04-01

**Authors:** Eva-Maria Weick, Peter Sarkies, Nicola Silva, Ron A. Chen, Sylviane M.M. Moss, Amy C. Cording, Julie Ahringer, Enrique Martinez-Perez, Eric A. Miska

**Affiliations:** 1 Wellcome Trust Cancer Research UK Gurdon Institute, Department of Genetics, University of Cambridge, Cambridge CB2 1QN, United Kingdom;; 2 MRC Clinical Sciences Centre, Imperial College Faculty of Medicine, London W12 0NN, United Kingdom

**Keywords:** piRNA biogenesis, *C. elegans*, 21U-RNA, PRDE-1

## Abstract

Piwi-interacting RNAs (piRNA) are small regulatory RNAs with essential roles in maintaining genome integrity in animals and protists. Most *Caenorhabditis elegans* piRNAs are transcribed from two genomic clusters that likely contain thousands of individual transcription units; however, their biogenesis is not understood. Here we identify and characterize *prde-1* (piRNA silencing-defective) as the first essential *C. elegans* piRNA biogenesis gene. Analysis of *prde-1* provides the first direct evidence that piRNA precursors are 28- to 29-nucleotide (nt) RNAs initiating 2 nt upstream of mature piRNAs. PRDE-1 is a nuclear germline-expressed protein that localizes to chromosome IV. PRDE-1 is required specifically for the production of piRNA precursors from genomic loci containing an 8-nt upstream motif, the Ruby motif. The expression of a second class of motif-independent piRNAs is unaffected in *prde-1* mutants. We exploited this finding to determine the targets of the motif-independent class of piRNAs. Together, our data provide new insights into both the biogenesis and function of piRNAs in gene regulation.

## Abstract (precis)

Piwi-interacting RNAs (piRNAs) are small regulatory RNAs with essential roles in genomic maintenance. Weick et al. identify *prde-1* as a novel *C. elegans* piRNA biogenesis gene. PRDE-1 is required specifically for the production of piRNA precursors from genomic loci containing an upstream motif, the Ruby motif. Intriguingly, the expression of a second class of motif-independent piRNAs is unaffected in *prde-1* mutants. This study defines a new paradigm for two distinct classes of piRNAs and provides insights into the biogenesis and function of piRNAs in gene regulation.

Piwi-interacting RNAs (piRNAs) have an evolutionarily conserved role in maintaining the genetic and epigenetic integrity of the germline in many organisms (Malone and Hannon 2009). piRNAs in ciliates are involved in genome editing (Chalker and Yao 2011), and in planaria, piRNAs are involved in regeneration and neoblast function (Reddien et al. 2005; Palakodeti et al. 2008). Their role in maintenance of fertility through transposable element silencing in the germline has been extensively studied. In *Drosphila* and mammals, piRNAs are produced from long precursor transcripts, which subsequently undergo processing to mature piRNA sequences with a length distribution of 24–32 nucleotides (nt) and a strong preference for a 5′ uracil. In conjunction with their Piwi protein partners, these small RNAs can target transposons by base-pairing and instigate secondary amplification cycles to ensure robust silencing of repetitive elements.

piRNAs are also conserved in the nematode *Caenorhabditis elegans* (Ruby et al. 2006; Batista et al. 2008; Das et al. 2008; Wang and Reinke 2008; Bagijn et al. 2012). *C. elegans* encodes two Piwi clade Argonaute (AGO) superfamily proteins, PRG-1 and PRG-2, although PRG-2 has likely little or no function (Batista et al. 2008; Das et al. 2008; Bagijn et al. 2012). Although piRNAs are slightly shorter in *C. elegans*, with a uniform length of 21 nt, they display a preference for a 5′ uracil similar to that observed in other animals. *C. elegans* piRNAs also have a 5′ monophosphate and a 3′ hydroxyl group and are post-transcriptionally modified by 2′O-methylation at the 3′-most nucleotide via the methyltransferase HENN-1 (Ruby et al. 2006; Billi et al. 2012; Kamminga et al. 2012; Montgomery et al. 2012). Mature piRNAs are absent in mutants lacking *prg-1*. They are restricted to the male and female germline, where they are required for normal fertility (Batista et al. 2008; Das et al. 2008; Bagijn et al. 2012).

Despite these similarities to piRNAs in other organisms, *C. elegans* piRNAs differ in both their mechanism of action and their production: *C. elegans* piRNAs silence transposons and protein-coding genes in a manner that is independent of Piwi endonuclease activity or “slicing.” Instead, *C. elegans* piRNAs silence transcripts in *trans* and often through imperfectly complementary sites by initiating a localized secondary endogenous siRNA (endo-siRNA) response (Bagijn et al. 2012; Lee et al. 2012). Secondary endo-siRNAs represent the most abundant class of endogenous small RNAs in *C. elegans*, are RNA-dependent RNA polymerase products, have a 5′ triphosphate, and are predominantly 22 nt in length with a 5′ guanine (22G-RNAs) (Sijen et al. 2001). piRNA-mediated silencing via an endo-siRNA pathway involves cytoplasmic factors such as the Mutator proteins MUT-7, MUT-2, and MUT-16; the RNA-dependent RNA polymerases RRF-1 and EGO-1; and the nuclear 22G-RNA Argonaute protein HRDE-1 as well as chromatin factors (Ashe et al. 2012; Buckley et al. 2012).

Endogenous protein-coding gene and transposon transcripts exhibit Piwi-dependent endo-siRNAs (22G-RNAs) at sites complementary to piRNAs and are derepressed in Piwi mutants. Intriguingly, piRNA-mediated silencing can establish a multigenerational silencing memory in the *C. elegans* germline that becomes independent of *prg-1* (Ashe et al. 2012; Luteijn et al. 2012; Shirayama et al. 2012). The significance of the ability of piRNAs to target genes in such a manner remains poorly understood, as do the rules that determine which genes become targets of the piRNA pathway and which remain independent.

piRNA biogenesis and maturation factors identified in *Drosophila* or mammals (Olivieri et al. 2010, 2012; Ipsaro et al. 2012; Nishimasu et al. 2012; Preall et al. 2012; Li et al. 2013) often have no clear ortholog in *C. elegans*. *C. elegans* piRNAs derive from two large clusters on chromosome IV (Ruby et al. 2006). However, unlike *Drosophila* or mammalian piRNA clusters, *C. elegans* piRNA clusters are interspersed with protein-coding genes. Within these clusters, ∼16,000 piRNAs are located on both strands with respect to genes and are intergenic or intronic but largely excluded from coding regions (Ruby et al. 2006; Batista et al. 2008; Bagijn et al. 2012). Several lines of evidence suggest that *C. elegans* piRNAs are derived from individual smaller transcription units rather than a long primary precursor: First, piRNA loci are associated with a sequence motif containing an 8-nt core consensus sequence, CTGTTTCA (Ruby et al. 2006), which we refer to here as the “Ruby motif.” This motif is located ∼40 base pairs (bp) upstream of the 5′ uracil of the piRNA with an A/T-rich spacer sequence. Ruby et al. (2006) postulated that this motif is part of a piRNA promoter motif. Second, consistent with this hypothesis, individual piRNAs can be expressed from short transgenes containing a single piRNA locus (Cecere et al. 2012; Billi et al. 2013). Third, a number of Forkhead family transcription factors can associate with the Ruby motif, and knockdown of these transcription factors results in a reduction in piRNA levels. Fourth, using 5′ RACE or CAP-selective sequencing, putative piRNA precursors of ∼70 nt (21UR-3372 and 21UR-14222) (Cecere et al. 2012) or ∼26 nt (genome-wide) (Gu et al. 2012) have recently been identified. Both studies suggest that piRNA precursors have a 2-nt 5′ sequence extension as compared with the mature 21U-RNA and are likely made by RNA polymerase II (Pol II). Taken together, these data support a model in which generation of short, capped, piRNA precursors is driven from the conserved Ruby motif. Interestingly, CAP sequencing also uncovered a novel subset of so-called type II piRNAs that associate with PRG-1 and are not derived from the piRNA clusters on chromosome IV, implying that PRG-1 itself does not select for piRNAs derived from Ruby motif loci (Gu et al. 2012).

Despite these recent advances into the understanding of the mechanism of *C. elegans* piRNA biogenesis, many aspects remain mysterious. In particular, our knowledge of the life of piRNAs before they engage with PRG-1 as well as whether different routes that piRNAs may take to enter a PRG-1/piRNA complex have any functional consequences is hampered by the lack of any factor other than *prg-1* that is essential for mature piRNA abundance. Here, using a forward genetic screen, we identified *prde-1* (piRNA silencing-defective) as the first such factor. We show that *prde-1* is a nuclear factor associated with piRNA clusters on chromosome IV defining the site of piRNA precursor generation. Intriguingly, *prde-1* is specifically required for the accumulation of both mature piRNAs and their precursors that come from genomic loci containing a Ruby motif. piRNAs that do not come from loci associated with a Ruby motif are not affected in *prde-1* mutants. Thus, we used *prde-1* mutants as a tool to assess the different targets of motif-dependent and motif-independent piRNAs. Together, our results clarify the distinct stages of piRNA biogenesis in *C. elegans* and extend classification of piRNAs and their targets.

## Results

### PRDE-1 is a novel protein required for Piwi/piRNA function

**Figure 1.**
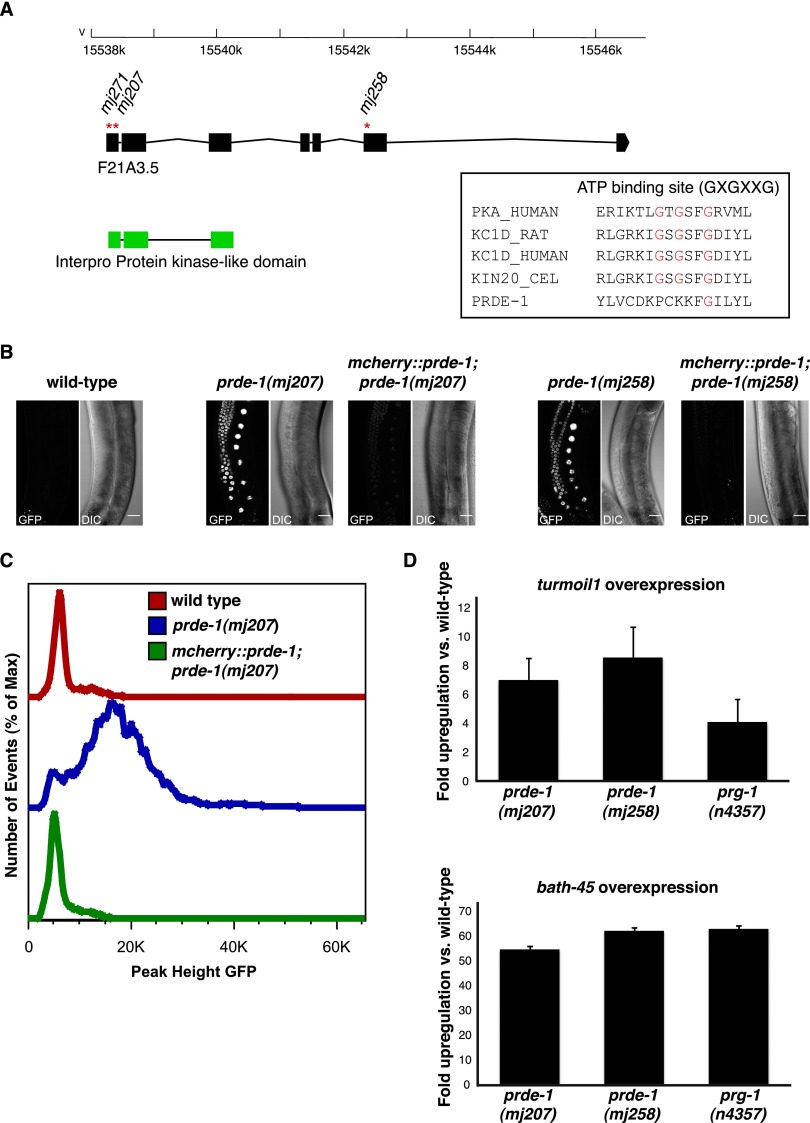
PRDE-1 is required for piRNA function. (*A*) Schematic representation of *prde-1* (F21A3.5) as in WormBase WS238. Red asterisks indicate the positions of stop alleles isolated in a mutagenesis screen for piRNA pathway mutants (see also Supplemental Fig. S1A). Exons encoding a predicted kinase domain in the transcript are shown as green boxes. A globin-like gene, *glb-14* (F21A3.6), encoded on the opposite strand is omitted for clarity. The *inset* shows clustal alignment of the ATP-binding sites of human protein kinase A, rat casein kinase δ, human casein kinase δ, and the *C. elegans* casein kinase *kin-20* (sequences downloaded from Uniprot) to the corresponding residues in PRDE-1. (*B*) Confocal images of piRNA sensor expression in wild type (*left*) and *prde-1* mutants and corresponding rescue lines (*middle* and *right*). Images are live specimens at 40× magnification; bars, 20 µm. (*C*) Flow cytometry analysis comparing GFP signal in the wild-type sensor (red), *prde-1(mj207)* (blue), and the corresponding rescue strain (green). (*D*) qRT–PCR of *turmoil1*, a transposable element piRNA target (*top* panel), and *bath-45*, a protein-coding target (*bottom* panel), in *prg-1* and *prde-1* mutants. Data are fold up-regulation relative to wild type. Error bars are SEM.

Previously, we developed an in vivo assay for piRNA function in the germline of *C. elegans* using a “piRNA sensor” transgene expressing a histone-GFP fusion protein in the germline that is responsive to the endogenous piRNA 21UR-1. In a wild-type background, the sensor transgene is efficiently silenced, whereas in mutants of piRNA pathway factors, such as *prg-1* and, for example, *mutator* class genes, the GFP transgene is derepressed (Supplemental Fig. S1A; Ashe et al. 2012; Bagijn et al. 2012). Using chemical mutagenesis, we performed a forward genetic screen to identify mutations that failed to silence the piRNA sensor (Supplemental Fig. S1B; Ashe et al. 2012). Three independent mutations from this screen, *mj207*, *mj258*, and *mj271*, mapped to the same region on chromosome V and failed to complement each other. Using genome resequencing, we determined that *mj258*, *mj207*, and *mj271* all had independent nonsense mutations in the uncharacterized predicted ORF F21A3.5 (Fig. 1A). We therefore named F21A3.5 *prde-1*. Sequence similarity searches using a number of different algorithms suggests that PRDE-1 has homology with kinase domains in the N-terminal part of the protein, with the closest matches being in the casein kinase family (Fig. 1A; Shi et al. 2001; Kelley and Sternberg 2009; Hunter et al. 2012). However, sequence alignment suggests that the ATP-binding site required for kinase activity (Longenecker et al. 1996; Endicott et al. 2012) is not conserved in PRDE-1 (Fig. 1A). PRDE-1 is conserved within the *Caenorhabditae*, including *Caenorhabditis briggsae* and *Caenorhabditis japonica*; however, we were unable to identify a clear ortholog of PRDE-1 in other animals.

To demonstrate that loss of *prde-1* is indeed responsible for piRNA sensor desilencing, we generated transgenic animals expressing an mCherry-PRDE-1 fusion protein in the germline and crossed these into *prde-1* mutant strains. While the piRNA sensor is desilenced in *prde-1(mj207)* and *prde-1(mj258)* mutants, expression of the mCherry-PRDE-1 fusion protein largely restored piRNA sensor silencing, as assessed by microscopy and flow cytometry (Fig. 1B,C). We concluded that PRDE-1 is required for piRNA sensor silencing. Next, we tested whether PRDE-1 was required for not only the silencing of our artificial piRNA sensor but also endogenous piRNA targets. Using *piwi/prg-1* mutants, we and others previously identified a number of piRNA targets in *C. elegans*, including mobile elements and protein-coding genes (Batista et al. 2008; Das et al. 2008; Bagijn et al. 2012; Lee et al. 2012). We therefore determined the expression of the transposable element *turmoil1* and the protein-coding gene *bath-45* using quantitative RT–PCR (qRT–PCR) in *prde-1* mutants. We found that both of these piRNA targets were up-regulated in *prde-1* mutants relative to wild type to an extent similar to in *prg-1* mutants (Fig. 1D). Together, these data suggest that PRDE-1 is a novel factor required for piRNA function.

### PRDE-1 is required for normal fertility

**Figure 2.**
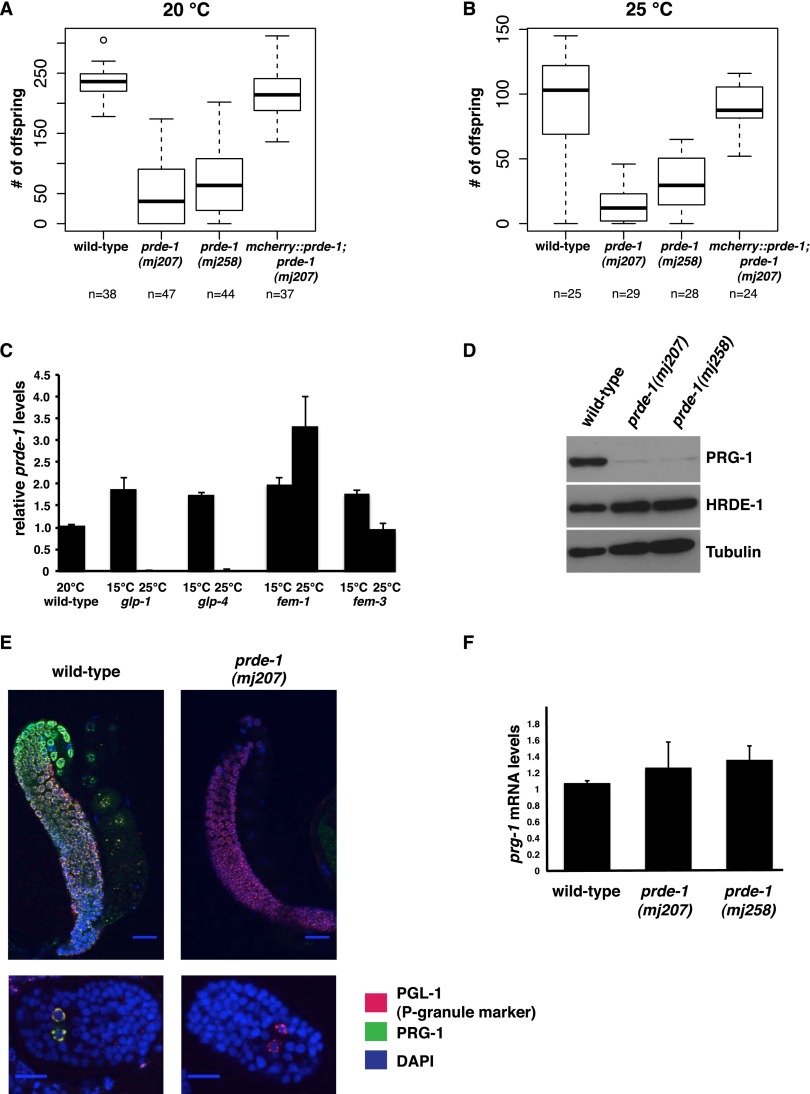
PRDE-1 is a nuclear germline protein required for normal fertility. (*A*,*B*) Progeny counts in wild-type and *prde-1* mutant animals and one *prde-1* rescue line at 20°C and 25°C. (*n*) Number of parental adults used (see Supplemental Fig. S2A,B). (*C*) *prde-1* is expressed in the male and female germline of *C. elegans*. qRT–PCR depicts changes in *prde-1* mRNA levels in temperature-sensitive germline mutants at the permissive (15°C) restrictive (25°C) temperatures. At the restrictive temperature, *glp-1* and *glp-4* are germline-less, *fem-1* is feminized, and *fem-3* is masculinized. Transcript levels are normalized to *actin-3*. For Ct = undetermined, values were set to the maximum Ct of 40. Error bars are the SEM of two biological replicates. (*D*, *top*) Western blot showing down-regulation of PRG-1 in *prde-1* mutants as compared with wild-type. HRDE-1, a germline-expressed nuclear Argonaute protein, is shown as a control for the presence of the germline (*middle*), and tubulin is shown as a general loading control (*bottom*). (*E*) Immunofluorescence staining of wild-type and *prde-1(mj207)* mutant animals for PRG-1 (green), the P-granule marker PGL-1 (OIC1D4) (red), and DNA (DAPI, blue). Distal germlines are shown on *top* (40× magnification; bar, 20 µm), and embryos with primordial germ cells are at the *bottom* (60× magnification; bar, 10 µm). (*F*) qRT–PCR of *prg-1* mRNA levels in wild-type and *prde-1* mutant animals. Data are normalized to the germline-expressed gene *cgh-1*. Error bars are SD; data are relative to a wild-type standard curve.

*prg-1* mutants display a range of fertility defects and have a reduced brood size (Batista et al. 2008; Das et al. 2008). Scoring *prde-1* mutant families at 20°C or 25°C, we found that the number of offspring is severely reduced as compared with wild type or *prde-1* mutants carrying the *mcherry∷prde-1* rescue transgene (Fig. 2A,B). These data are similar to what has been observed for *prg-1* (Batista et al. 2008; Das et al. 2008; Wang and Reinke 2008). In addition, we observed a small degree of embryonic lethality in both *prde-1* alleles analyzed (data not shown). Finally, maintaining *prde-1* mutant families for successive generations in the laboratory, we noticed instances of complete sterility where adult animals were devoid of embryos (Supplemental Fig. S2A,B). These data support a prominent role for PRDE-1 in germline integrity in *C. elegans*.

### PRDE-1 expression is restricted to the germline

As *prg-1* expression is restricted to the germline of *C. elegans*, we wanted to test whether *prde-1* displays a similar expression pattern. We took advantage of a set of temperature-sensitive mutants that affect germline development at the restrictive temperature (25°C) (Fig. 2C). *prde-1* mRNA was readily detectable via qRT–PCR in extracts from wild-type animals kept at 20°C and was present at similar levels in the different mutant backgrounds at the permissive temperature (15°C). In contrast, at the restrictive temperature, *prde-1* mRNA was not detected from *glp-4(bn2*ts*)* and *glp-1(e2144*lf*)* mutant animals, which are devoid of germ cells (Beanan and Strome 1992). However, *prde-1* mRNA was still detected in RNA from *fem-1(hc17*ts*)* (Nelson et al. 1978; Kimble et al. 1984) and *fem-3(q22*sd,ts*)* (Barton et al. 1987) mutants at the restrictive temperature that are devoid of sperm or oocytes, respectively. Thus, *prde-1* is exclusively expressed in the male and female germline of *C. elegans*.

### PRDE-1 is not a general RNAi factor

**Figure 3.**
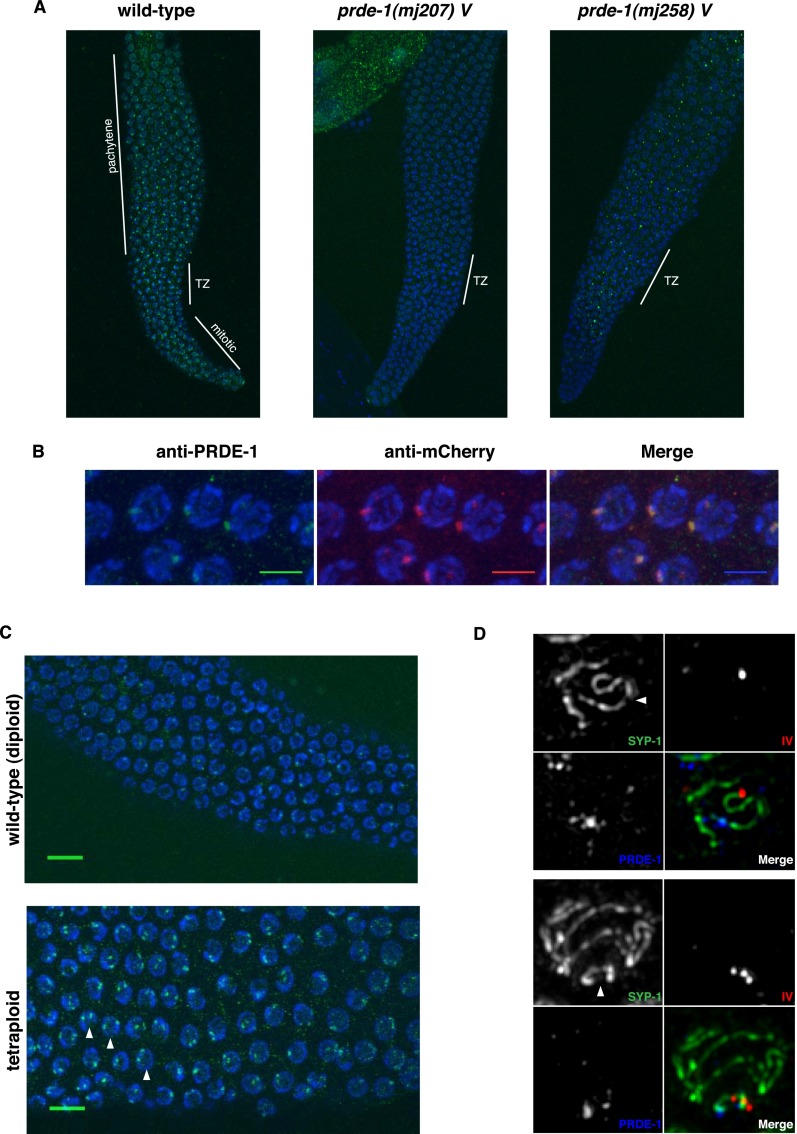
PRDE-1 is a nuclear factor associated with sites of piRNA biogenesis. (*A*) Immunofluorescence of endogenous PRDE-1 in isolated distal gonads of *C. elegans*. Images are Z-projections (20× magnification; 3.0 zoom), with two putative PRDE-1-null alleles shown for comparison. Distinct regions of the *C. elegans* germline are marked by white lines. (TZ) Transition zone. (*B*) Confocal Z-projections of pachytene germ cell nuclei in an mCherry-PRDE-1-expressing strain stained for PRDE-1 and mCherry (60× magnification; 10× zoom; bars, 5 µm). (*C*) Immunofluorescence of endogenous PRDE-1 in pachytene nuclei of wild-type diploid (*top*) and tetraploid (*bottom*) *C. elegans* (confocal Z-projections at 40× magnification; 3.2 zoom). Arrowheads indicate prominent examples of nuclei with two PRDE-1 foci. Bars, 10 µm. (*D*) Representative deconvolved wide-field fluorescence images of pachytene nuclei stained for synaptonemal complex marker SYP-1 and PRDE-1 in combination, with DNA-FISH probe T21D12 marking the left end of chromosome IV. The *top* panel set shows partial projection of one nucleus, and the *bottom* panel set shows Z-projection of a complete nucleus. Arrowheads point to chromosome IV.

Early studies exploring *C. elegans* small RNA pathways demonstrated that genes required for transposon silencing are often also involved in RNAi-related phenomena (Ketting et al. 1999; Tabara et al. 1999). This is the case for many of the Mutator class genes (e.g., *mut-7* and *mut-2*), which act downstream from *prg-1* in the piRNA pathway (Bagijn et al. 2012), are effective suppressors of germline transposition (Mut) (Ketting et al. 1999), and are defective in exogenous (environmental) RNAi (Rde) (Tabara et al. 1999). In contrast, *prde-1* mutants are as sensitive to RNAi as wild-type animals, while *mut-*2 mutants are not, as assessed by feeding with a somatic and a germline RNAi trigger (Supplemental Fig. S2C,D). These data in conjunction with the localization data shown below in Figure 3 suggest that PRDE-1 function is distinct from that of other general RNAi factors. In addition, *prde-1* mutants are also competent in heritable RNAi, whereas the downstream Piwi/piRNA pathway gene *hrde-1* is not (Ashe et al. 2012; data not shown).

### PRDE-1 acts upstream of PRG-1

To place PRDE-1 within the Piwi/piRNA pathway, we next considered whether PRDE-1 and PRG-1 are required for the localization or stability of each other. PRG-1 protein levels are strongly reduced in *prde-1* mutants when assayed by Western blotting from whole adult extracts (Fig. 2D), a finding that we further confirmed using immunohistochemistry: PRG-1 is not detectable using an anti-PRG-1 antibody in the adult germline and primordial germ cells in the embryo (Fig. 2E). Importantly, costaining with a P-granule marker suggests that P granules remain intact in *prde-1* mutant animals. *prg-1* transcript levels are comparable with wild type in *prde-1* mutant animals (Fig. 2F). Moreover, a GFP-PRG-1 fusion protein expressed from a heterologous promoter (*mex-5*) promoter (schematic in Supplemental Fig. S2E, left panel) (Bagijn et al. 2012) shows a similar reduction in expression in *prde-1* mutants (Supplemental Fig. S2F). Thus, reduction in PRG-1 levels in prde-1 mutants is likely to be a post-transcriptional effect. Interestingly, any residual GFP-PRG-1 still detectable in *prde-1* mutants after image processing still appears to be localized correctly to P granules. Together, these data suggest that PRDE-1 is directly or indirectly required for PRG-1 protein stability. In contrast, the mCherry-PRDE-1 fusion protein expressed from a heterologous promoter (schematic in Supplemental Fig. S2E, right panel) remains unchanged in expression level and localization in *prg-1* mutant animals as compared with animals carrying a rescuing GFP-PRG-1 transgene (Supplemental Fig. S2F). We therefore concluded that PRDE-1 acts upstream of PRG-1.

### PRDE-1 localizes to nuclear foci associated with chromosome IV

Next, we assessed the localization of PRDE-1 protein within the germline. Previous studies have shown that PRG-1 is localized to perinuclear P granules in the syncytial germ cytoplasm (Batista et al. 2008; Wang and Reinke 2008; Ashe et al. 2012). Downstream endo-siRNA factors, required for piRNA function, such as MUT-7, are localized adjacent to P granules in cytoplasmic “mutator foci” (Phillips et al. 2012), whereas factors mediating transcriptional silencing of piRNA targets, such as the HRDE-1 Argonaute protein, are nuclear (Ashe et al. 2012). Using an antibody against the endogenous protein, we observed a striking pattern of speckles absent in *prde-1* mutant animals in the distal germline (Fig. 3A). High-resolution microscopy with the PRDE-1 antibody as well as the mCherry-PRDE-1 transgene revealed that the majority of PRDE-1 signal forms a single defined focus within nuclei of the pachytene germline (Fig. 3B; Supplemental Fig. S3A). While the majority of nuclei within the pachytene region, where homologous chromosome pairs are aligned for crossing over, contain one prominent focus, we sometimes observed two spots in the more distal mitotic region of the germline, where chromosomes have not entered prophase I of meiosis yet (data not shown). This led us to speculate that PRDE-1 may be associated with one particular chromosome pair. Supporting this idea, pachytene nuclei in the germline of tetraploid worms (Madl and Herman 1979) displayed two rather than one prominent PRDE-1 focus (Fig. 3C). We thus hypothesized that PRDE-1 may be associated with the piRNA clusters on chromosome IV, the site of the majority of piRNA loci in *C. elegans* (Ruby et al. 2006; Batista et al. 2008, Das et al. 2008). To test this, we used DNA FISH staining of chromosome IV in combination with PRDE-1 and SYP-1 immunostaining. As the FISH probe used targets the left end of chromosome IV and the piRNA clusters are found on the far right and central part of the chromosome, use of the synaptonemal complex component SYP-1 allowed for tracing of the entire chromosome length (MacQueen et al. 2002). Our data show that PRDE-1 is indeed associated with chromosome IV distal to the FISH probe signal in proximity of the piRNA clusters (Fig. 3D).

### PRDE-1 is essential for piRNA/21U-RNA biogenesis or stability

**Figure 4.**
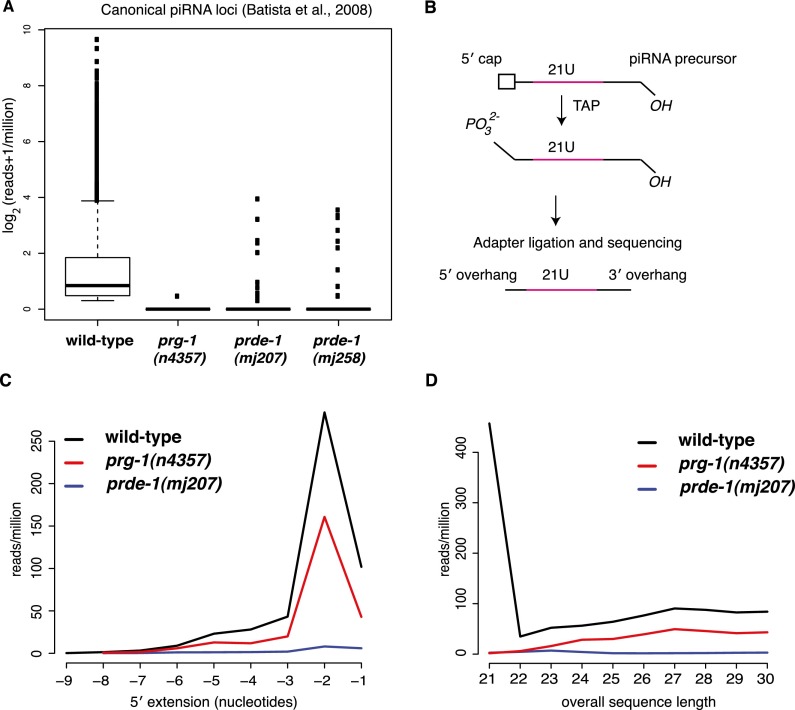
PRDE-1 is required for piRNA precursor biogenesis. (*A*) 21U small RNA reads mapping perfectly to known *C. elegans* piRNA loci, as annotated previously (Batista et al. 2008), in *prde-1* mutants compared with *prg-1*. 5′-dependent small RNA libraries were prepared from wild-type, *prg-1(n4357)*, *prde-1(mj207)*, and *prde-1(mj258)* RNA. Boxes represent interquartile range, with the median indicated by a line, and they extend to the maximum point no more than 1.5-fold greater than the interquartile range. Outliers are indicated by dots. (*B*) Outline of strategy for detecting potential piRNA precursors by deep sequencing. (*C*) Distribution of 5′ extending nucleotides for sequences mapping to piRNA sequences as used in *A* but that are >21 nt in wild type (black line), *prg-1* (red line), and *prde-1(mj207)* (blue line). These are likely capped piRNA precursors. (*D*) Overall length of all sequences, which map perfectly to piRNA loci (i.e., either 21 nt in length or above) in wild type, *prg-1*, and *prde-1*. Color code is as in *C*.

Given the distinct localization of PRG-1 and PRDE-1, a direct effect of PRDE-1 on PRG-1 protein stability seemed unlikely. Therefore, we considered the possibility that PRDE-1 was acting instead on the small RNA component of the PRG-1 complex (Batista et al. 2008), the piRNAs (21U-RNAs), thereby affecting PRG-1 stability indirectly. Our findings regarding the association of PRDE-1 with chromosome IV strongly supported this postulated role for PRDE-1 in piRNA biogenesis. To characterize piRNA populations, we performed 5′ monophosphate-dependent small RNA sequencing from wild-type and mutant animals and searched for sequences that matched to previously annotated 21U-RNA sequences (Batista et al. 2008). As expected, piRNAs were almost completely eliminated in mutants lacking *prg-1* (*P* < 2 × 10^−16^, Wilcox test for reduction relative to wild type). Similarly, piRNAs were also absent in two different *prde-1* mutant lines (*P* < 2 × 10^−16^, Wilcox Test for reduction relative to wild type) (Fig. 4A). We confirmed this result by Northern blotting (Supplemental Fig. S4A) and further showed that piRNAs are restored in *prde-1* mutants upon introduction of the rescue Cherry-PRDE-1 rescue transgene (Supplemental Fig. S4B). Thus, we concluded that PRDE-1 acts as an upstream piRNA biogenesis factor.

### PRDE-1 is required for piRNA/21U-RNA precursor biogenesis

The reduction in piRNA abundance in *prde-1* mutants could reflect a role in either piRNA stability or the generation of piRNAs. Recently, evidence has suggested that individual piRNA loci in *C. elegans* are transcribed separately by RNA Pol II to produce individual capped long piRNA precursors, which are subsequently processed into mature piRNAs (Cecere et al. 2012; Gu et al. 2012). We therefore treated total RNA with tobacco acid pyrophosphatase (TAP) to remove 5′ caps, thus allowing cloning of potential piRNA precursors (Fig. 4B). We found a strong enrichment for a 2-nt extension at the 5′ end, in agreement with recent data (Gu et al. 2012), suggesting that our protocol successfully identified putative piRNA precursor sequences. Furthermore, the length distribution of precursors suggested that the majority of sequences were <40 nt, although slightly longer than those reported by Gu et al. (2012) (Fig. 4B; Supplemental Fig. S4C,D). To confirm that these sequences are indeed capped and thus Pol II products, we first isolated nuclear RNA and then performed additional stringent enzymatic purification, including polyphosphatase and terminator exonuclease treatment, to remove any other contaminating RNAs prior to TAP cloning of capped RNAs. In contrast to libraries prepared from total RNA, mature piRNA sequences of 21 nt in length were depleted in this library. Consistent with our previous results, we detected longer sequences showing a strong enrichment for a 5′ extension of 2 nt. These sequences showed a distribution of lengths that peaked between 28 and 29 nt, with very few sequences >36 bp long (Supplemental Fig. S4E,F). Thus, two independent techniques converge on the idea that piRNA precursors in *C. elegans* are made in the nucleus, have a 5′ cap, and carry a 2-nt extension at the 5′ end.

Interestingly, the piRNA precursor sequences were still present in *prg-1* (Fig. 4C,D; Supplemental Fig. S4E,F) despite complete absence of the mature 21U sequences themselves, supporting the idea that these species are produced independently of *prg-1* activity. In *prde-1* mutants, however, capped piRNA precursors from the set of canonical piRNA loci were almost completely absent. The few remaining sequences did not show the prominent 2-nt extension at the 5′ end and did not display the same overall length distribution as the precursor sequences in wild-type and *prg-1* mutant libraries (Fig. 4C,D). Equally, we did not detect the precursor sequences in the capped nuclear sequences in *prde-1* animals (Supplemental Fig. S4E,F). As mature piRNAs are absent in *prde-1*, this loss of capped, 5′-extended sequences provides for the first time strong genetic evidence that these species are indeed the first intermediate product of generation of mature 21U-RNAs. Moreover, based on the length profile of precursors detected (Supplemental Fig. S4D,F), the presence of any longer precursor species is unlikely. In conclusion, our data show that PRDE-1 is required for the biogenesis of piRNA precursors.

### PRDE-1 is exclusively required for the biogenesis of piRNAs with an upstream Ruby motif

**Figure 5.**
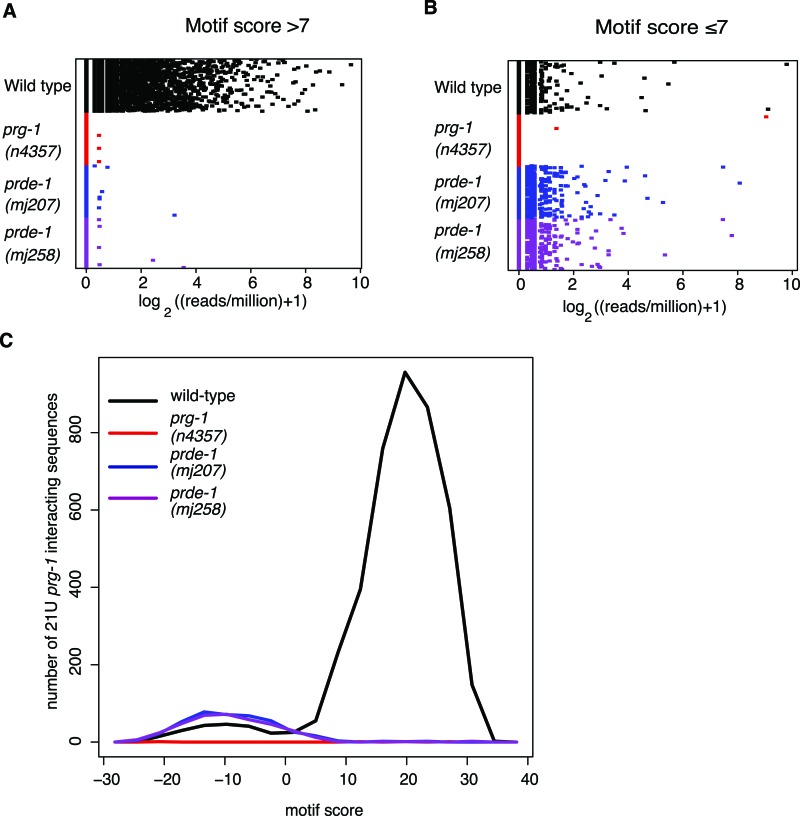
PRDE-1 is specifically required for Ruby motif-dependent piRNAs. (*A*,*B*) Dot chart showing read count for each *prg-1*-interacting sequence, as identified in both Batista et al. (2008) and Gu et al. (2012). *A* shows PRG-1-interacting small RNAs from loci where the motif score is >7, calculated as described elsewhere (Ruby et al. 2006; Batista et al. 2008), while *B* shows PRG-1-interacting small RNAs that come from loci where the motif score is ≤7. (*C*) Distribution of motif scores among all prg-1-interacting sequences in wild type, *prg-1*, and *prde-1*. The *X*-axis is the midpoint of a window of width 5 U, and the *Y*-axis shows counts for unique sequences with motif scores within the window.

As biogenesis of classical piRNAs in *C. elegans* is dependent on an upstream Ruby motif (Ruby et al. 2006; Batista et al. 2008), we wondered whether PRDE-1 might be involved in the selection of piRNA loci. Initial characterization of piRNAs used a scoring matrix weighted using the upstream regions of PRG-1-interacting sequences to define a motif score of >7 as the cutoff for a piRNA locus (Ruby et al. 2006; Batista et al. 2008). Recently, deeper sequencing of PRG-1-interacting small RNAs identified an extended list of potential 21U-RNA sequences, a subset of which were not dependent on the upstream motif (Gu et al. 2012). We therefore examined whether these sequences were present in our deep-sequencing data sets. We combined the new PRG-1-interacting sequences with those defined previously (Batista et al. 2008) and recalculated the motif score according to the method defined previously (Ruby et al. 2006). We grouped this extended data set into two classes: motif-dependent (motif score >7) and motif-independent (motif score ≤7). Both of these classes of 21U-RNAs were well represented in libraries from wild-type animals, whereas both classes of 21U-RNAs were completely absent in *prg-1* deep-sequencing data, suggesting that both motif-dependent and motif-independent 21U-RNAs depend on PRG-1 for their stability (Fig. 5A). In *prde-1* mutant animals, we found that the new 21U-RNAs with a motif score of >7 were completely absent (Fig. 5A), recapitulating our earlier finding that PRDE-1 is required for the presence of piRNAs (Fig. 4A). Strikingly, however, we observed that the new 21U-RNAs with a motif score of ≤7 were still present in *prde-1* mutants and indeed showed slightly increased levels relative to wild type (Fig. 5B). In addition, piRNA precursor sequences from motif-independent piRNAs were also still present in *prde-1* mutant animals and have the same 2-nt 5′ extension found for motif-dependent precursors (Supplemental Fig. S5A). Taken together with the absence of 21U-RNAs defined as having a motif score of >7 (Fig. 4A), this suggests that PRDE-1 is only required for biogenesis of piRNAs that are produced from loci associated with the Ruby motif. This is consistent with our data regarding the localization of PRDE-1 on the right arm of chromosome IV, as this region contains the highest density of Ruby motifs in the genome (Ruby et al. 2006; Batista et al. 2008; Das et al. 2008). In addition, the smaller number of motif-dependent piRNAs that are derived from loci not on chromosome IV were also absent in *prde-1* mutants (Supplemental Fig. S5B). On the other hand, piRNAs that are motif-independent can be produced without PRDE-1 activity. Indeed, analysis of the motif scores for the extended set of 21U-RNAs showed a bimodal distribution of motif scores in the wild type, indicative of two distinct populations of piRNAs. The motif scores for 21U-RNAs present in *prde-1* mutants showed only a single peak with a modal motif score of −10 (Fig. 5C). Based on these data, we propose a modified classification of *C. elegans* piRNAs into Ruby motif-dependent (and *prde-1*-dependent) and motif-independent (and *prde-1*-independent) piRNAs.

### Analysis of prde-1 mutants reveals endogenous targets of motif-independent piRNA targets

**Figure 6.**
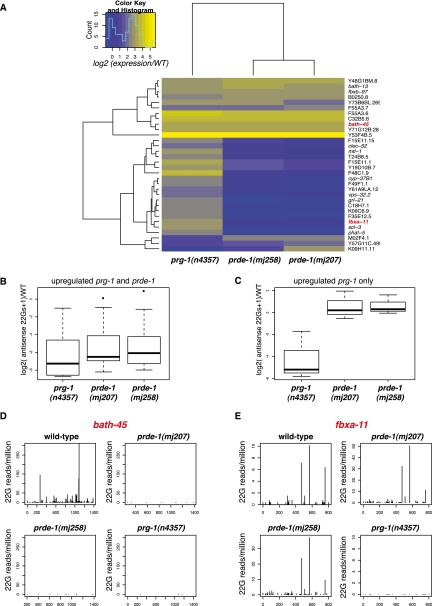
PRDE-1-dependent and -independent piRNAs target a distinct set of genes. (*A*) Heat map showing the behavior of all genes that are statistically significantly up-regulated by more than fourfold with a *P* < 0.05, with a multiple test correction *P* < 0.1, in at least one out of *prg-1* and the two *prde-1* alleles relative to wild type. The color of each entry in the heat map shows the difference in mean expression relative to wild type (WT) as seen in the color key. (*B*,*C*) 22G-RNAs mapping to genes up-regulated in either *prg-1* and *prde-1* (*B*) or *prg-1* alone (*C*). The number of antisense 22Gs relative to wild type, normalized to total library size, is shown on the *Y*-axis. Boxes represent interquartile range, with the median indicated by a line, and they extend to the maximum point no more than 1.5-fold greater than the interquartile range. Outliers are indicated by dots. (*D*,*E*) 22G-RNAs mapping to representative target genes of *prde-1*-dependent (*D*) and *prde-1*-independent (*E*) piRNAs. The start position of each 22G-RNA is plotted on the *X*-axis, with number of reads normalized to total library size on the *Y*-axis.

As Ruby motif-dependent and motif-independent piRNAs differ in their requirement for PRDE-1, we attempted to identify possible functions of motif-independent piRNAs through analysis of gene expression changes in *prg-1* and *prde-1* mutants. We identified genes that were up-regulated by more than fourfold (*P* < 0.05, Student's *t*-test) in *prg-1* mutants or either of two independent *prde-1* alleles relative to wild type using microarray expression analysis (Fig. 6A). The majority of these genes fell into two categories: one up-regulated in both *prde-1* and *prg-1* mutants and one up-regulated in *prg-1* mutants only. Only one gene was statistically significantly up-regulated in *prde-1* alone, a statistically significant depletion compared with the other categories (*P* < 0.01, Fisher's exact test). Known piRNA target genes such as *bath-45* (Bagijn et al. 2012) were found in the set of genes up-regulated in both *prg-1* and *prde-1*. Extending these sets to genes with greater than twofold increases, *prde-1*-independent *prg-1*-regulated genes displayed a distinct pattern of gene ontology enrichment as compared with *prde-1*-dependent genes (Supplemental Table S4). *C. elegans* innate immune genes (Sinha et al. 2012) were significantly (*P* < 0.05, Fisher's exact test) overrepresented in *prde-1*-independent *prg-1*-regulated genes (Supplemental Fig. S6A). Moreover, gene set enrichment analysis using the entire array data confirmed statistically highly significant up-regulation of innate immunity genes in *prg-1* relative to both wild type and *prde-1* (permutation estimated *P* < 0.01) (Supplemental Fig. S6B). We therefore hypothesized that the different sets of genes up-regulated in *prg-1* versus *prde-1* mutants might reflect Ruby motif-dependent and motif-independent piRNA targets. Sixty-four percent of genes up-regulated in both *prg-1* and *prde-1* and 59% of genes up-regulated only in *prg-1* were targeted by at least one piRNA, allowing up to three mismatches, compared with 47% of all genes (Fisher's exact test *P* = 0.1 and *P* = 0.03, respectively). However, using direct sequence matching to known piRNA sequences is likely an insensitive method to identify possible targets (Bagijn et al. 2012). Instead, loss of 22G siRNAs generated at putative piRNA target sites in *prg-1* can be used for target prediction (Bagijn et al. 2012). Overall, changes in *prg-1* and *prde-1* 22G-RNA levels relative to wild type correlated well (Supplemental Fig. S6C,D). Both *prg-1*-specific and *prg-1*/*prde-1* shared genes had robust levels of antisense 22G-RNAs mapping to them in wild type, although, interestingly, *prg-1*-specific genes had fewer 22G-RNA reads (*P* < 0.05, Wilcoxon unpaired test) (Supplemental Fig. S6E). Both categories showed strong reductions in 22G levels in *prg-1*. However, in *prde-1* mutants, *prg-1*/*prde-1* shared genes showed clear reductions in the 22G-RNA level (Fig. 6B), but 22G-RNAs mapping to *prg-1*-specific genes were unaltered (Fig. 6C, individual examples in D,E). Thus, Ruby motif-independent piRNAs act similarly to motif-dependent piRNAs, producing 22G-RNAs that lead to target gene silencing.

## Discussion

Here we identified a first gene, *prde-1*, that is essential for the production of piRNA precursors upstream of PRG-1 in *C. elegans*. Our characterization of the defects in piRNA biogenesis in *prde-1* mutants has provided new insights into both the mechanism of piRNA biogenesis and the function of piRNAs in *C. elegans*.

### A model for piRNA biogenesis in *C. elegans*

**Figure 7.**
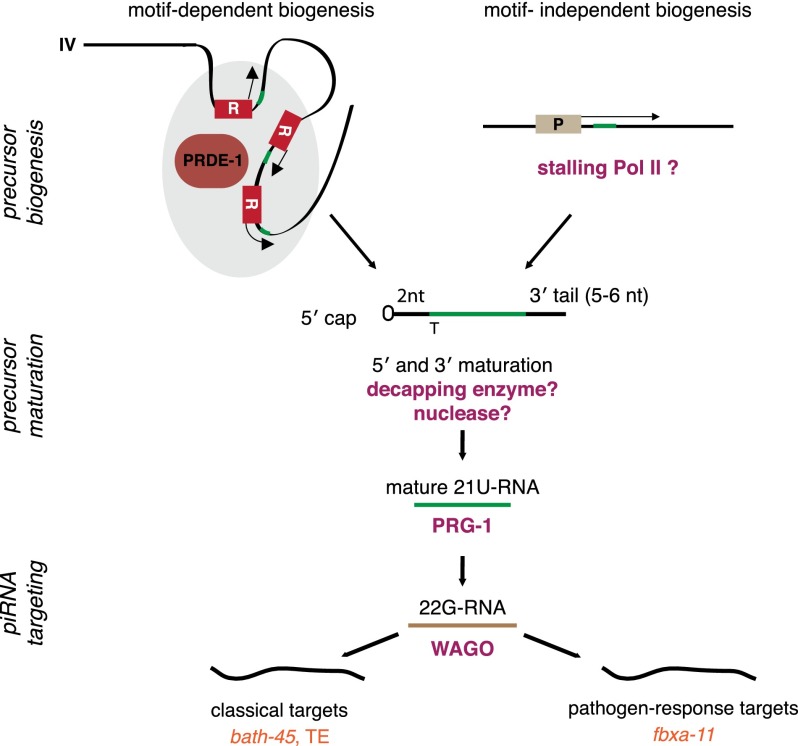
A model of piRNA biogenesis in *C. elegans*. piRNA precursor generation occurs either downstream from the conserved Ruby motif in a *prde-1*-dependent manner or independent of the motif and *prde-1*, possibly as a consequence of RNA Pol II stalling during transcription. (R) Ruby motif; (P) genic promoter; (gray shading) foci of piRNA biogenesis defined by PRDE-1; (green) 21U-RNA sequences. In this model, Ruby motif loci outside the canonical piRNA clusters on chromosome IV may either come in contact with this major biogenesis site or accumulate PRDE-1 at sites distal to chromosome IV. Precursors are capped, begin 2 nt upstream of the mature 21U-RNA sequence, and are 28–29 nt long. Maturation occurs by an unknown process, possibly involving decapping enzymes and nucleases. Mature 21U-RNAs are incorporated into PRG-1 and recognize targets by imperfect complementary base-pairing. This process occurs in perinuclear P granules and leads to recruitment of factors, such as RNA-dependent RNA polymerases and Mutator class proteins (not shown), required for generation of downstream 22G-RNAs. These in turn bind to worm-specific Argonaute proteins (WAGO) (for example, HRDE-1) and mediate target silencing in the nucleus or possibly the cytoplasm. Classical Ruby motif-dependent piRNAs target protein-coding genes and transposable elements, whereas motif-independent piRNAs have targets enriched for pathogen response genes.

Our data support a hierarchical model for piRNA biogenesis in *C. elegans* (Fig. 7). First, piRNA precursors are transcribed by RNA Pol II from genomic loci either with the Ruby motif or from motif-independent loci. PRDE-1 is essential for piRNA precursors originating from piRNA loci with the Ruby motif, and piRNA precursors from motif-independent loci are unaffected in *prde-1* mutant animals (Fig. 5). Although we cannot exclude the possibility that PRDE-1 is required for stability or processing of precursors specifically arising from motif-containing loci, the simplest mode of PRDE-1 action would be upstream of the production of a Ruby motif-dependent precursor. A role for PRDE-1 very early in piRNA biogenesis is strongly supported by our in vivo data showing that PRDE-1 foci are associated with chromosome IV, the main site of piRNA loci (Fig. 3). Given that piRNAs produced from motifs not on chromosome IV are also dependent on PRDE-1 activity, PRDE-1 may associate with loci on other chromosomes, forming foci that are below the detection limit of our assays. It is also possible that PRDE-1 may itself define a piRNA production factory, which Ruby motif-containing loci could move into. Further work will be required to clarify the exact mechanism by which PRDE-1 promotes piRNA precursor formation at the sites of Ruby motifs; however, our findings provide an exciting starting point for further in-depth investigation of piRNA biogenesis.

Our analysis suggests that piRNA precursors produced by RNA Pol II transcription from both Ruby motif-dependent and motif-independent loci have a 5′ cap and a 2-nt 5′ extension. This is in agreement with a previously reported genome-wide analysis of capped sequences (Gu et al. 2012). However, there has been some debate over the length of piRNA precursors: Cecere et al. (2012) found ∼70-nt-long precursors, while Gu et al. (2012) have reported precursors of ∼26-nt sequence length. The length distribution that we found sequencing both total short RNAs up to 40 nt and nuclear RNAs up to 100 nt was different from that reported by Gu et al. (2012), with a modal length of 28 nt rather than 26 nt (Supplemental Fig. S4D,F). Nevertheless, based on our data, we can clearly conclude that piRNA precursors are predominantly <36 nt. How the transcription machinery is regulated to give rise to such short sequences at piRNA loci will be an interesting question to address.

The second stage of piRNA biogenesis involves the maturation of piRNA precursors, culminating in the incorporation of mature 21U-RNAs into a PRG-1 complex. This stage is dependent on PRG-1 and is likely uncoupled from the production of precursors in the nucleus, as we show that *prg-1* itself is not required for the production of piRNA precursors from either motif-independent or motif-dependent loci (Fig. 4C; Supplemental Fig. S4E,F). Furthermore, in agreement with data showing that *prg-1* is able to bind to both motif-dependent and motif-independent piRNAs (Gu et al. 2012), we show that mature piRNAs from both Ruby motif-dependent and motif-independent loci are completely absent in *prg-1* mutants (Fig. 5A,B). This suggests that *prg-1* is unable to discriminate between the two types of loci and thus likely acts downstream from piRNA precursor production.

A role for PRG-1 downstream from the production of piRNA precursors is also supported by our data showing a reduction in PRG-1 protein levels in *prde-1* mutants. The absence of motif-dependent piRNAs in *prde-1* mutants would mean that PRG-1 is incompletely loaded and thus, as has been shown for Piwi in *Drosophila* (Olivieri et al. 2010), will be unstable. The small amount of residual PRG-1 present in *prde-1* is correctly localized and likely represents PRG-1 bound to motif-independent piRNAs, consistent with the small proportion of motif-independent piRNAs within the overall piRNA pool (see Fig. 5C).

### A functional classification of piRNAs in *C. elegans*

Recently, the discovery of PRG-1-interacting 21U-RNAs that do not derive from the piRNA clusters on chromosome IV in *C. elegans* was used to propose a new classification of piRNAs (type I and type II) (Gu et al. 2012). Our classification of piRNAs into Ruby motif-independent and motif-dependent refines this idea, as the “type II” piRNAs include piRNAs with high-scoring motifs that were not on chromosome IV. We found that piRNAs with high-scoring motifs that were not on chromosome IV were nevertheless absent in *prde-1* mutants (Supplemental Fig. S5C), arguing that the motif itself is more important in differentiating between classes of piRNAs. This supports recent work showing that a transgene containing the piRNA motif inserted on chromosome II could nevertheless produce robust levels of piRNAs (Billi et al. 2013).

piRNAs that are not produced from the Ruby motif are generally present at lower abundance than motif-dependent piRNAs. It was therefore conceivable that they are simply capped breakdown products of the transcription of genes that are incorporated into the PRG-1 pathway perhaps by chance (Gu et al. 2012). However, our data for the first time demonstrate that Ruby motif-independent piRNAs are functional: They have target genes that are up-regulated in *prg-1* mutants but not in *prde-1* mutants and, moreover, are able to produce 22G-RNAs at their target sites (Fig. 6). Thus, Ruby motif-independent piRNAs regulate target genes through a downstream secondary siRNA pathway, as we reported previously for Ruby motif-dependent piRNAs (Bagijn et al. 2012).

### The role of Ruby motif and non-Ruby motif piRNAs in gene regulation

Our analysis of the targets of Ruby motif-dependent and motif-independent piRNAs shows that piRNAs from the two types of genomic loci tend to target different types of genes. Since the mechanism of silencing appears to be the same for both types of piRNAs, different sequence properties of the piRNAs themselves may be important. One possible distinction between motif-dependent and motif-independent piRNAs is that motif-independent piRNAs derived from transcribed protein-coding genes may be under more sequence constraint than the motif-dependent piRNAs. However, the target genes of motif-independent piRNAs appear to be enriched for innate immunity genes (Supplemental Fig. S6A,B), which evolve extremely rapidly in *C. elegans* (Maydan et al. 2010). Indeed, target genes of both motif-independent and motif-dependent piRNAs evolve more rapidly than the median for protein-coding genes (*P* < 0.05, Wilcox unpaired test) (Supplemental Fig. S7). Thus, there might be selective pressure on genes to avoid Ruby motif-independent piRNAs, and a “snapshot” of these targets reveals only the genes that move most rapidly through the sequence space. Alternatively, the ability of the piRNA pathway to target fast-evolving genes may be beneficial; for example, by contributing to the fine-tuning of expression levels of newly evolved genes.

While the enforced repression of pathogen response genes in the germline may be puzzling at first, it is conceivable that such genes need to be repressed in normal environmental conditions but may be derepressed upon immunogenic insult. Indeed, down-regulation of somatic small RNA pathways upon infection can lead to relief of repression of pathogen response genes (Kudlow et al. 2012; Sarkies et al. 2013). A similar mechanism may be acting in the germline mediated by the piRNA pathway.

Overall, our examination of *prde-1* mutants has confirmed the importance of both Ruby motif-dependent and motif-independent piRNAs in gene expression control in *C. elegans*. This study provides a starting point for a more detailed examination of the evolutionary history of this ancient yet extraordinarily dynamic component of the animal kingdom's genome defense armory.

## Materials and methods

### Genetics

*C. elegans* were grown under standard conditions (Brenner 1974) at 20°C unless otherwise indicated using *Escherichia coli* strain HB101 as a food source (*Caenorhabditis* Genetics Center, University of Minnesota, Twin Cities, MN). All strains used are listed in Supplemental Table S1. Additional standard *C. elegans* experimental procedures, including RNAi and progeny counts, are detailed in the Supplemental Material.

### Transgenics

The piRNA sensor and GFP-PRG-1 lines were described previously (Bagijn et al. 2012). The pDONR P4-P1R mex-5 promoter cherry construct (pJA281) was a gift from Julie Ahringer's laboratory (Zeiser et al. 2011). For the *mcherry∷prde-1* fusion construct, we cloned F21A3.5 from N2 cDNA, including the STOP codon (1581 nt total) into pDONR221. The par-5 3′ untranslated region (UTR) was generated from N2 genomic DNA (655 nt) and inserted into pDONR-P2R-P3. The sequence of all pDONR constructs was confirmed by sequencing. To generate transgenic animals, germline transformation was performed as described (Mello and Fire 1995). Injection mixes contained 2–20 ng/µL MosSCI plasmid and 5–10 ng/µL marker plasmid DNA (see the Supplemental Material for details). Single-copy transgenes were generated by transposase-mediated integration (MosSCI) as described (Frøkjaer-Jensen et al. 2008, 2012).

### piRNA sensor EMS screen

After EMS treatment following standard protocols (Brenner 1974), F2 or F3 offspring of mutagenized worms were sorted using a Copas Biosort large-particle sorter as described in Bagijn et al. (2012). Further details are as described previously (Ashe et al. 2012). Chromosome mapping and genotyping of mutations are described in the Supplemental Material.

### Immunostaining, FISH, and imaging

Extensive procedures (including antibodies) for imaging of live specimen and immunostaining of isolated gonads using confocal microscopy as well as information on FISH in combination with wide-field fluorescence imaging are provided in the Supplemental Material.

### Protein extraction and Western blotting

Synchronized populations of worms were grown on 90-mm NGM agar plates to the gravid adult stage, and generation of protein lysates and Western blotting were performed according to standard procedures. The primary antibodies used were custom rabbit anti-PRG-1 (1:1000) (Kamminga et al. 2012), rabbit anti-HRDE-1 (1:4000) (Ashe et al. 2012), and monoclonal mouse anti-α-tubulin clone DM1A (1:10,000; Sigma-Aldrich). The secondary antibodies used were ECL anti-mouse IgG HRP from sheep and ECL anti-rabbit IgG HRP from donkey (both GE Healthcare). For visualization of bands, we used Immobilon Western chemiluminescent HRP substrate (Millipore).

### RNA extraction

Worms synchronized by L1 starvation arrest were grown on 90-mm plates to the required stage, washed several times in M9 buffer, snap-frozen in TRISure (Bioline) at a ratio of 1 vol of worms to 10 vol of TRISure, and cracked open by five freezing–thawing cycles in liquid nitrogen. RNA extraction was performed using standard procedures.

### qRT–PCR

qRT–PCR experiments and oligonucleotides for qRT–PCR are described in the Supplemental Material (Supplemental Table S3).

### Microarray

RNA was extracted as described above from each of the following strains in three biological replicates: wild-type piRNA sensor (SX1316), piRNA sensor; *prde-1(mj207)* (SX2470), piRNA sensor; *prde-1(mj258)* (SX2471) and *prg-1(n4357)*; and piRNA sensor (SX1888). RNA samples (4 × 3) were used to generate cDNA libraries followed by microarray hybridization. Sample processing from total purified RNA and Affymetrix *C. elegans* 1.0 gene arrays were performed by the EMBL Genomic Core Facilities (Heidelberg, Germany). Data were processed using the Affymetrix Expression console. Processed microarray data are available in comma-separated table format (.csv) as Supplemental Data File 1. A description of further data analysis is in the Supplemental Material.

### Small RNA sequencing

Five micrograms of total RNA was pretreated with either 20 U of TAP (Epicentre) or 20 U of RNA 5′ polyphosphatase (Epicentre) in a volume of 20 µL for 45 min at 37°C or used directly. After standard extraction, RNA was used for TruSeq small RNA library preparation according to the manufacturer's instructions (Illumina), including 15 cycles of PCR. Libraries were sequenced on a MiSeq instrument (Illumina). A detailed description of processing and analysis of the data thus generated are in the Supplemental Material.

Purification of nuclear short RNAs was performed as described (Chen et al. 2013). Briefly, nuclei of 2 million young adults grown in liquid culture were isolated as described in Ooi et al. (2010), and RNA was extracted using Tripure (Roche). Short capRNA-seq libraries were cloned from 20 µg of nuclear RNA as follows: After size selection for RNAs of 20–100 nt, we performed RNA polyphosphatase (Epibio) treatment followed by Terminator exonuclease (Epibio) treatment and 3′ adapter ligation. After treatment with heat labile alkaline phosphatase (Epibio), capped RNAs were rendered accessible for cloning by TAP treatment. 5′-adapter cloning and library generation were completed as described in the TruSeq small RNA kit (Illumina), and sequencing was performed on a HiSeq instrument (Illumina, SE50).

### Accession numbers

The sequencing data reported in this study have been deposited in the Gene Expression Omnibus (GEO) database (http://www.ncbi.nlm.nih.gov/geo) under the accession number GSE49220.
